# Cationic Bioactive Peptide from the Seeds of *Benincasa hispida*

**DOI:** 10.1155/2014/156060

**Published:** 2014-04-16

**Authors:** Sunayana Sharma, Hirday Narain Verma, Nilesh Kumar Sharma

**Affiliations:** ^1^School of Life Sciences, Jaipur National University, Jaipur 302025, India; ^2^Department of Molecular Biology and Genetics, Dr. D.Y. Patil Vidyapeeth, Dr. D. Y. Patil Biotechnology and Bioinformatics Institute, Mumbai-Bangalore Highway, Tathawade, Pune 411033, India

## Abstract

A designated bioactive peptide “Hispidalin” purified from the seeds of *Benincasa hispida*, which is a medicinal plant, belongs to Cucurbitaceae family. Purification was achieved by using a procedure consisting of extraction from potassium phosphate buffer followed by FPLC and HPLC steps. Based on amino acid residue, this peptide is amphipathic and basic with one net positive charge having isoelectric pH 8.1. This peptide is without sulphur containing amino acid suggesting its extended conformation lacking double bond secondary structure. The results obtained from MALDI-TOF suggested that Hispidalin is of molecular mass 5.7 KDa with 49 amino acid residues and confirmed SDS-PAGE resolved **∼**6.0 KDa protein band. This novel and unknown peptide “Hispidalin” showed broad and potent inhibitory effects against various human bacterial and fungal pathogens; its growth inhibition was significantly comparable with commercial antibacterial and antifungal drugs. The Hispidalin at 40 **μ**g/mL concentration exhibited 70.8% DPPH free radical-scavenging activity and 69.5% lipid peroxide inhibition. Thus, in the present study, Hispidalin demonstrated remarkable antimicrobial and antioxidant potentials from the seeds of *B. hispida*.

## 1. Introduction

Plants are one of the major sources of peptide. Potentially, peptides have considerable medical importance since they [1] affect the stability and sensory quality of plant foods [2]. Research on bioactive proteins/peptide has been increasing including work on the development of pathogen resistant and antimicrobial compounds [2–6]. In recent years, extensive scientific evidence has been provided for the existence of biological active peptides and proteins derived from plants that might have beneficial effects upon human health [6–12]. Peptides have certain biological activities like antimicrobial and antioxidant activities [2, 4, 6, 13]. Several plants in the family Cucurbitaceae have been widely used as medicine in many countries of Asia including India.* B. hispida* commonly known as wax guard has been used in treatment of gastrointestinal problems [14], antidepressant-like activity [15], antinociceptive, antipyretic [16], anticompulsive effects [17], and antimicrobial activity [18]. Plants are rich in a wide variety of protein that has found antimicrobial properties [1, 2, 6, 11, 19]. Plants do not have an immune system directly comparable with that of animals. Thus, to protect themselves from infection by a variety of pathogens, plants have evolved a host of defense mechanisms [2, 4, 12, 19]. In recent decades, a number of antimicrobial peptides (AMPs) have been identified or predicted from various organisms including plant sources. AMPs in general consist of 10–50 amino acid residues [2, 13, 19]. These peptides do not have any specific consensus amino acid sequences that are responsible for their biological activity, but most of them maintain certain common features, such as containing positive charge and relatively hydrophobic and amphipathic structure. Antimicrobial proteins are produced by many organisms including vertebrates, invertebrates, plants, and fungi [5, 12, 20, 21]. They serve to protect the organisms from pathogenic bacteria and fungus, which would bring devastating damage. Several plant proteins capable of inhibiting the growth of agronomically important pathogens have been isolated during the last few years [4, 8, 20]. Cationic peptides vary considerably in sequence and structure, with a few common features. Cationic peptides are amphipathic meaning they possess both a hydrophobic region that interacts with lipids and a positively charged hydrophilic region that interacts with water or negatively charged residues [3, 4, 8, 19, 22]. This feature allows the peptides to interact well with membranes that are composed of amphipathic molecules, especially negatively charged bacterial membranes. For the most part, animal cells tend to have membranes with no net charge so they are unaffected by cationic peptides [5, 12, 21]. The findings of several studies have evidenced that protein/peptide from animal and plant proteins can act as direct scavengers of diverse free radicals or behave as antioxidants in model systems [9, 12, 23–25]. In recent years, the antioxidant activities of proteins/peptides hydrolysates from plant-derived proteins, including* Sphenostylis stenocarpa* [23], hemp seed [9], phaseolin and bean [24], and* Jatropha curcas *[26], have been evaluated using several* in vitro* antioxidant evaluation systems such as diphenyl-1-picryhydradzyl (DPPH) and linoleic acid oxidation. The antioxidant properties of these peptides largely depend on the peptide structure, amino acid composition, and their molecular mass [9, 23, 24, 26]. In the present findings, we have attempted to report first unknown peptide designated as Hispidalin from* B. hispida* seeds displaying remarkable and promising antimicrobial and antioxidant activity. Hispidalin primary structure differs from all other known plant proteins. The findings will lead to development of bioactive peptide having broad application in pharmaceutical and therapeutic industry.

## 2. Material and Method

### 2.1. Biological Materials

The fresh fruit of* B. hispida* is collected from Agra city of Uttar Pradesh in January 2009. Seeds were separated from fruit and oven-dried at 40°C for 48 h. All clinical isolates of bacteria and fungus were obtained from patients at the Microbiology Department, SMS Hospital Jaipur.

### 2.2. Isolation Procedure

Five hundred gram seeds of* B. hispida *were crushed in the mortar pastel; paste was made with acetone and left covered overnight (16 h) at normal temperature, then suspended in extraction buffer I of 20 mM potassium phosphate buffer, pH 6.5, 5.0 mM EDTA, and 1.0 mM DTT for 3 h, and centrifuged 10000 rpm for 10 min at 4°C. The supernatant containing soluble proteins was collected and designated fraction I. The pellet was resuspended in extraction buffer I, centrifugation step was repeated, and the supernatants were pooled. The remaining pellet was resuspended in extraction buffer II of 20 mM potassium phosphate buffer, pH 6.5 containing EDTA, DTT, and urea in concentrations of 2 mM, 1 mM, and 4 M, respectively, with 2% Triton X 100. Further, it was homogenized three times and centrifuged at 10,000 ×g for 10 min at 4°C. The supernatant was collected and mixed with fraction I. Thereafter, 80% ammonium sulphate was added to fat free fraction for ageing of precipitate for 12 h, and formed precipitate was collected through Whatman filter paper and stored in tube already containing 20 mM potassium phosphate buffer, pH 6.5.

### 2.3. Purification

Precipitate was dialyzed and centrifuged at 10000 rpm for 10 min maintaining temperature at 4°C. Supernatant was collected and filtered through 0.2 *μ*m syringe filter and then the filtrate was applied directly to column Fast Performance Liquid Chromatography (FPLC) Sephadex G-75 column. The column and pump were washed with milli-Q water, which is purified and deionized by Millipore water purification system using 0.2 *μ*m filter at flow rate of 0.5 mL/min and preequilibrated with 50 mM potassium phosphate buffer, pH 6.5. The separating 5 fractions were collected and concentrated by speed back vacuum concentrator. The fraction P5 showed maximum antimicrobial activity, which is determined by disc diffusion method, applied to HPLC (Water, BioSuite C-18 column, 4.6 mm × 250 mm). Column and pump were washed with milli Q water HPLC grade at the flow rate of 0.8 mL/min and then equilibrated with isopropanol HPLC grade at the flow adjusted to 0.8 mL/min at wavelength of 280 nm. Two fractions were separately collected through HPLC and further concentrated in speed back vacuum concentrator until the volume of sample reduced by one tenth of volume. The concentrated protein sample was tested for antimicrobial activity which is determined by disc diffusion method.

### 2.4. Polyacrylamide Gel Electrophoresis (SDS-PAGE) of Protein

The purity test and further analysis of purified peptide Hispidalin were performed using standard tricine SDS-PAGE with slight modifications [27].

### 2.5. MALDI-TOF/MS Peptide Characterization

The purified Hispidalin was submitted to MALDI-TOF (Bruker Daltonics) for the amino acid composition and peptide fingerprint analysis. The peptide was digested with trypsin and spectra were collected in monoisotopic negative ion mode.

### 2.6. Antimicrobial Activity

The bacterial strains used were* Escherichia coli*,* Pseudomonas aeruginosa*,* Bacillus cereus*,* Staphylococcus aureus,* and* Salmonella enterica* and the fungal strains were* Aspergillus flavus*,* Penicillium chrysogenum*,* Fusarium Solani*,* Colletotrichum gloeosporioides*, and* Curvularia geniculata*. These bacterial and fungal strains were all obtained as clinical isolates from patients at the Microbiology Department, SMS Hospital Jaipur. Stock cultures were maintained at 4°C on slant of nutrient agar (NA) for bacteria and potato dextrose agar (PDA) for fungus, prior to their use. Antimicrobial activity of peptide sample was evaluated by the paper disc diffusion method [28]. Nutrient agar plate was prepared for bacteria and potato dextrose agar plate was prepared for fungus 40 *μ*g/mL protein sample transferred to the 6 mm blank paper disc. Dried disc was placed on the plates previously inoculated with a bacterial suspension (concentration of 10^6^ cfu/mL) and fungus. The incubation condition maintained at 35°C for 24 h for bacteria and 7 days for fungus. Plates were then examined for the presence of growth inhibition zones, and diameters were measured, if any. Ciprofloxacin disc for bacteria was used as positive control and griseofulvin disc for fungus and water as negative control. The experiment was done in triplicate.

### 2.7. Total Protein Estimation

Total protein estimation was performed as per the method of Bradford (1976).

### 2.8. DPPH Free Radical Scavenging Activity Assay

The DPPH reagent was prepared by dissolving the 0.04 g DPPH to 95% of 100 mL methanol. Then the 2.95 mL of DPPH reagent was added to the 0.05 mL protein sample with varied concentrations (5, 10, 15, 20, 25, 30, 35, and 40 *μ*g/mL). Then, the absorbance of reaction mixture was measured at 517 nm using spectrophotometer (Varian Cary 100 UV Visible). *α*-Tocopherol (1 mg/mL) was used as standard and the DPPH without protein sample was used as control. Lower absorbance of the reaction mixture indicates higher free radical scavenging activity [29].

### 2.9. Ferric Thiocyanate- (FTC-) Thiobarbituric Acid (TBA) Method Based Lipid Peroxidation Determination

The standard method described by [30] was used with some modification. In brief, 40 *μ*g/mL protein samples was mixed with 4 mL of ethanol (99.5%). Then, the mixture added with 4.1 mL of 2.5% linolenic acid in 99.5% ethanol, 8.0 mL of 0.05 M potassium phosphate buffer, pH 7.0, and 3.9 mL of water was placed in a screw-capped vial and then placed in an oven at 40°C in the dark. Then, 0.1 mL of this solution was added to 9.7 mL of 75% ethanol and 0.01 mL of 30% ammonium thiocyanate. Further, 0.2 mL of 0.02 M ferrous chloride in 3.5% HCl was added to the mixture. At the end of the seven days, 2 mL of 20% tricholoacetic acid and 2 mL of 0.67% TBA were added to 1 mL of sample mixture prepared above. This mixture was then placed in boiling water bath at 100°C for 10 minutes. After cooling, it was centrifuged at 3000 ×rpm for 20 min at normal temperature. Then the absorbance of supernatant at 532 nm using spectrophotometer (Varian Cary 100, UV visible) was measured. The mixture without adding protein sample was used as control and *α*-tocopherol was used as the standard.

## 3. Results and Discussion

In nature, abundant and diverse antimicrobial peptides are produced by several organisms including invertebrate, plant, animal, and bacterial species [2, 12, 19–21]. Their amino acid composition, amphipathicity, cationic charge, and size allow them to be attached to and inserted into membrane bilayers to form pores by “barrel-stave,” “carpet,” or “toroidal-pore” mechanisms [2, 6, 12]. Most of the antimicrobial peptides identified are cationic peptides with low molecular weight and exhibit hydrophobic properties [2, 6, 12, 21]. Based on the three-dimensional structures of known peptides, antimicrobial peptides are generally classified into four major groups including alpha-helix, beta-sheet, loop, and extended peptides. It is believed that the amphipathic structure of antimicrobial peptides is essential to their antimicrobial activity [6, 10, 12, 31]. This Hispidalin is quite different from the defensine, a major class of cationic antimicrobial peptide from plants. Their sizes vary from 12–50 amino acids and have molecular masses of less than 10000 [6, 11, 12, 20, 32, 33]. Cationic peptides are amphipathic conveying that they have both a hydrophobic region that interacts with lipids and a positively charged hydrophilic region that interacts with water or negatively charged residues [6, 11, 12, 20, 32, 33]. This feature permits the peptides to intermingle well with membranes that are composed of amphipathic molecules, especially negatively charged bacterial membranes [6, 12]. For the most part, animal cells tend to have membranes with no net charge so they are unaffected by cationic peptides [6, 12, 20].

### 3.1. Extraction and Purification of Peptide from Seeds of* B. hispida*

**Figure 1:**
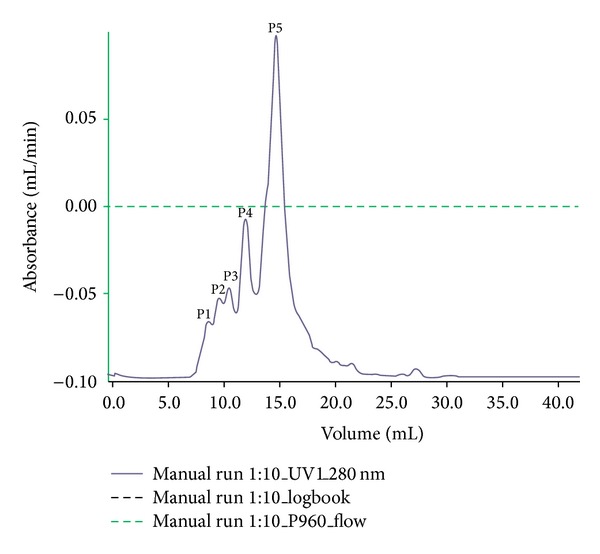
Sephadex G-75 Fast Performance Liquid Chromatography profile of antimicrobial peptide enriched fraction from* B. hispida* seeds. The monitoring of protein fraction was done at 280 nm wavelength and major peaks are represented as P1, P2, P3, P4, and P5.

**Figure 2:**
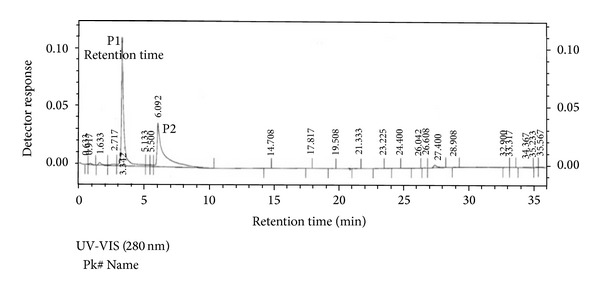
Reverse phase chromatography profile of the antimicrobial peptide enriched fraction P5 obtained from the FPLC purification presented in Figure 1. The elution profile was monitored at 280 nm UV-VIS detector. We identified two peaks P1 and P2 obtained from HPLC.

**Figure 3:**
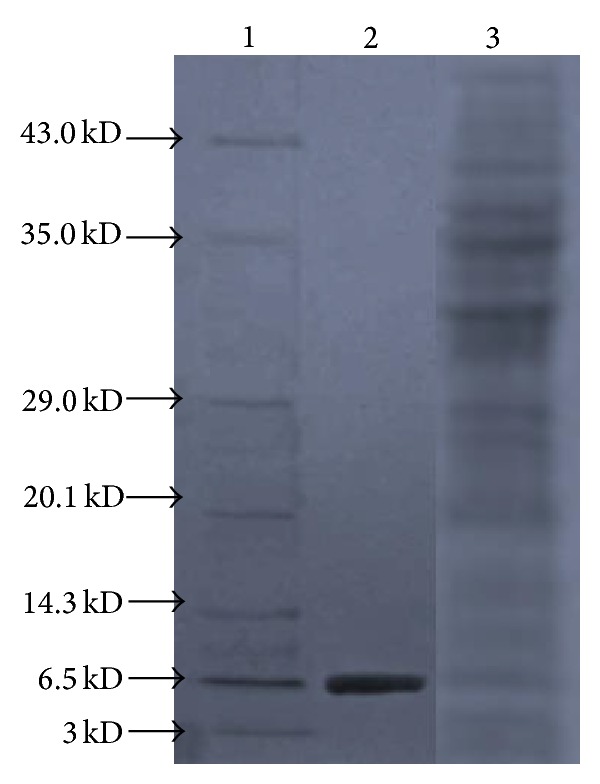
Tricine SDS-PAGE profile of Hispidalin peptide from* B. hispida*. Lane 1: low molecular weight protein marker, Lane 2: ten microgram of purified Hispidalin was loaded, and Lane 3: twenty microgram of Crude* B. hispida* seed protein was loaded.

**Figure 4:**
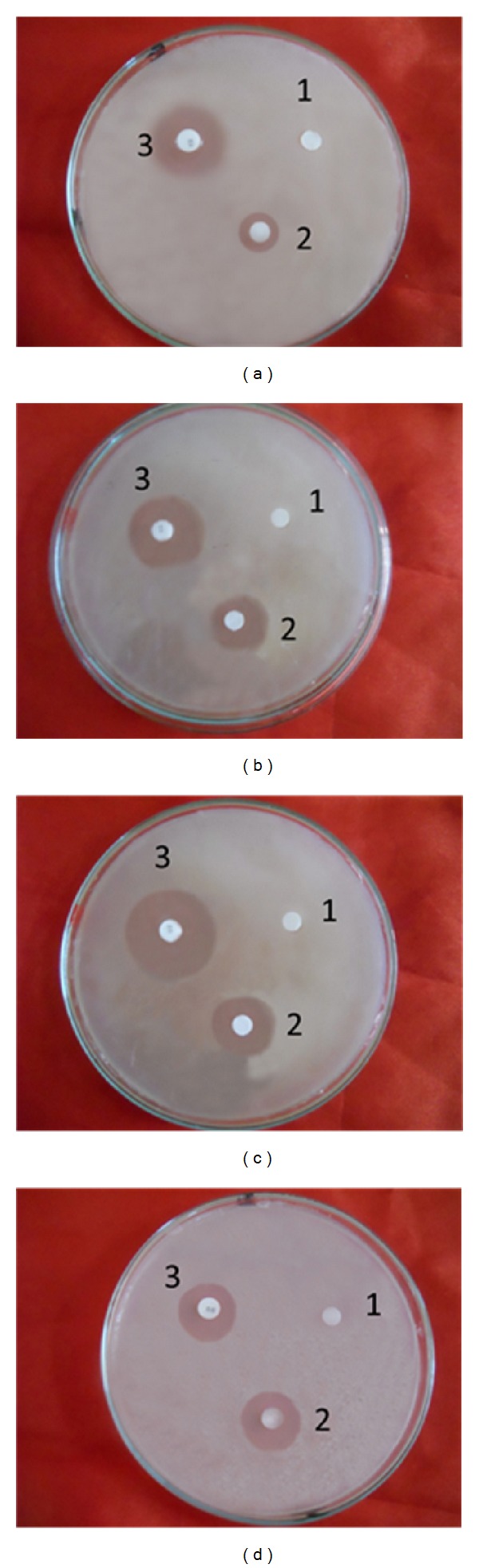
Antibacterial activity of bioactivity guided fraction from* B. hispida* against* E. coli* (a), fraction P4 from FPLC, (b) fraction P5 from FPLC, (c) fraction P1 from HPLC, and (d) fraction P2 from HPLC. 1: only water, 2: peptide, and 3: ciprofloxacin.

Our initial effort was to extract and isolate pure peptide from seeds of* B*.* hispida*. The weight of fruit taken was 3800 g and the weight of seeds extracted from fruit was 500 g. The weight of precipitate obtained from fraction after 80% ammonium sulphate precipitation was 38 g. After precipitation, the precipitate was applied to Sephadex G-75 column, which yielded five peaks (Figure 1) and purification yields are presented in Table 2. Out of which the P5 had maximum biological activities including antimicrobial and antioxidant activities shown in Table 1 and Figures 3(a) and 3(b). P5 fraction was submitted to final purification by HPLC, which yielded two P1 and P2 peaks (Figure 2) out of which the P1 had maximum biological activities including antimicrobial and antioxidant activity. As zone of inhibition determined by disc diffusion against* E. coli* growth was found to be 29 mm for HPLC-P1 presented in Table 1 and Figure 4, this fraction HPLC-P1 also demonstrated maximum DPPH scavenging activity up to 76.83% and 72.3% lipid peroxidation inhibition shown in Table 1. The fraction P1 was submitted to triacine-SDS-PAGE analysis to resolve its molecular weight. Based on gel analysis, we observed a single ~6.0 kDa peptide band depicted by (Figure 3), suggested to be of bioactive peptide of interest. Here, obtained bioactive peptide purified from* B. hispida* is designated as Hispidalin name.

### 3.2. MALDI-TO-TOF MS/MS Characterization of Hispidalin Peptide from* B. hispida* Seeds

**Figure 5:**
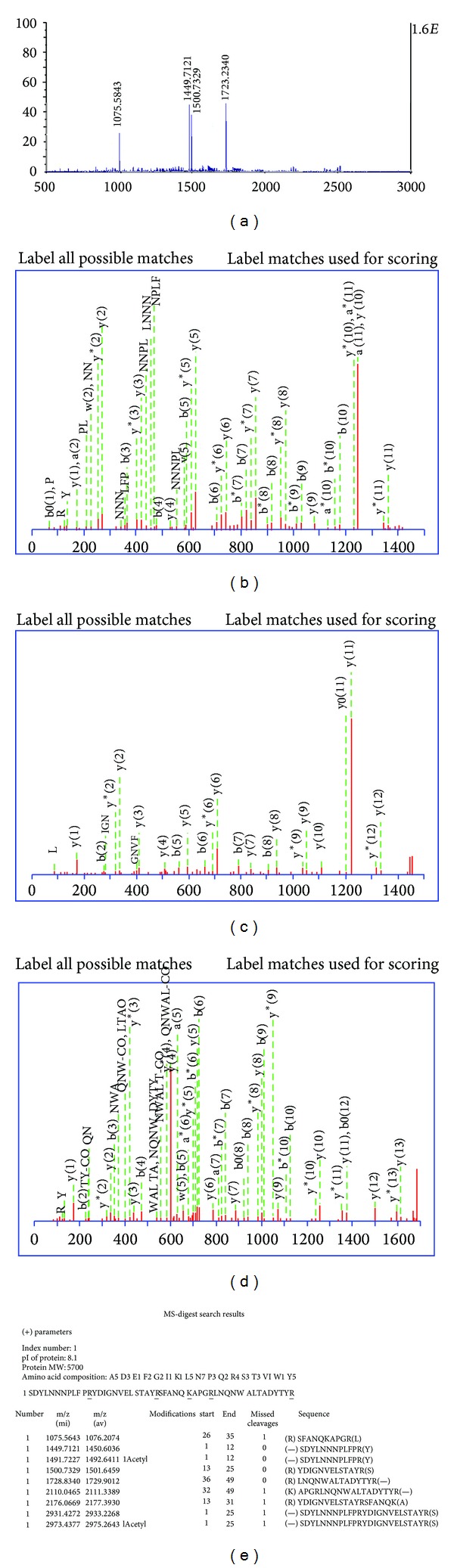
(a) MALDI-TOF MS spectra of trypsinized Hispidalin peptide. (b) MS/MS fragmentation of first peptide 1 from Hispidalin -SDYLNNNPLFPR using MALDI-TOF. (c) MS/MS fragmentation of Peptide 2 from Hispidalin YDIGNVELSTAYR using MALDI-TOF. (d) MS/MS fragmentation of peptide 3 from Hispidalin LNQNWALTADYTYR using MALDI-TOF. (e) MS-digest pattern of Hispidalin peptide amino acid sequence using the UCSF protein prospector software.

The MALDI-TOF-TOF MS/MS analysis of Hispidalin peptide was done to confirm the molecular weight and amino acid sequence analysis as determined by the Tricine-SDS-PAGE. In Figure 5(a), MALDI-TOF MS spectra of trypsinized Hispidalin is provided and it showed four different small peptides with molecular mass of peptide 1 (1075.56), peptide 2 (1449.7121), peptide 3 (1500.73), and peptide 4 (1728.83). We also performed the MS/MS analysis of the obtained peaks after trypsinization of the Hispidalin. The TOF-MS-MS spectra of each trypsinized smaller peptide consisting of a series of* y* and* b* ions and several ions are illustrated in Figures 5(a), 5(b), and 5(c). The MS-digest pattern of Hispidalin peptide amino acid sequence using the UCSF protein prospector software is also presented in Figure 5(d). The molecular weight of the peptide was determined to be 5.7 Kodak, with 49 amino acid sequences as follows: SDYLNNNPLFPRYDIGNVELSTAYRSFANQKAPGRLNQNWALTADYTYR. The amino acid sequence alignment was performed against the NCBI database and APD. During MALDI-TOF MS/MS analysis, the peptide generated four trypsin digested fragments having MW of peptide 1 (1075.56), peptide 2 (1449.7121), peptide 3 (1500.73), and peptide 4 (1728.83). We summed up MW of four peptides, it is around 5.7 KDa, which confirmed the Tricine-SDS-PAGE data showing ~6.0 KD in size depicted in Figures 5(a), 5(b), 5(c), and 5(d). The low similarity of the amino acid sequences indicated that the Hispidalin peptide is the first novel reported peptide from* B*.* hispida *seeds. This protein has 49 amino acid residues having both hydrophobic and hydrophilic residues. After analyzing the peptide sequence using mass analyzer, it indicates that this is cationic peptide with one net positive and having isoelectric pH-8.1. On the basis of one excess positively charged amino acid, this peptide has +1 net negative charge, which is in agreement that basic peptides has been reported to have +1 to +7 negative charge on their extended backbone. Important information about Hispidalin is that it does not contain any sulphur containing amino acid. Therefore, results strongly suggest that Hispidalin is without any folded structure and may exist in their native extended conformations. The amphiphilic nature of Hispidalin strengthens its candidacy to be a potent antimicrobial agent. Matrix-assisted laser desorption/ionization (MALDI) time-of-flight (TOF) mass spectrometry (MS) is now routinely used in many laboratories for the rapid and sensitive identification of proteins by peptide mass fingerprinting (PMF). When we submitted peptide sequence to NCBI database, it only matched hypothetical and uncharacterized protein of Molecular mass 23 KDa from* Aeromonas veronii* having maximum identity 66% [34]. It emphasizes our findings that this antimicrobial cationic peptide is new and there is no report from any organisms. To the best of our knowledge, this is the first information that basic low molecular weight peptide Hispidalin from* B. hispida* seeds demonstrated promising antimicrobial and antioxidant activity. We strongly believe that Hispidalin with 49 amino acid amino acid residue is a basic peptide with one net negative charge isolated from the seeds of* B. hispida*. At the same time, Hispidalin showed no sequence homology to any known plant protein, except with the hypothetical and uncharacterized protein from the bacteria. All cationic peptides are encoded in the form of bigger precursors with signal sequences that are later on modified by cleavage or group addition reactions such as glycosylation or halogenation [6, 12, 20].

### 3.3. Antimicrobial Activity of Hispidalin Peptide

We have assessed and demonstrated the potent antibacterial and antifungal activity of Hispidalin against selected bacterial and fungal strain.

### 3.4. Antibacterial Activity of Hispidalin

**Figure 6:**
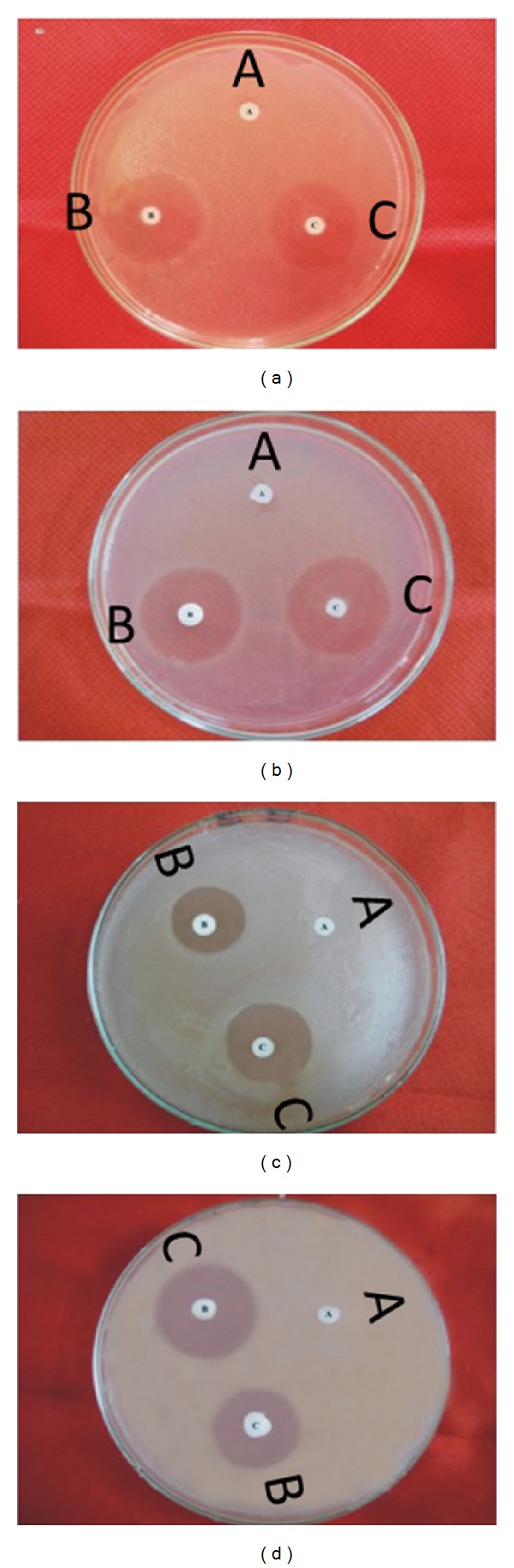
Antibacterial activity of Hispidalin (40 *μ*g/mL) peptide against* E. coli *(a), II-* S. enterica *(b),* P. aeruginosa *(c), and* S. aureus *(d). (A) For negative control (water), (B) positive control (ciprofloxacin), and (C) Hispidalin disc.

Antibacterial activity (assessed in terms of inhibition zone and activity index) of peptide by disc diffusion method tested against selected microorganisms were recorded and presented in (Figure 6 and Table 3). In the present study,* in vitro* antibacterial activity of Hispidalin have shown maximum zone of inhibition 29 mm against* S. enterica* and minimum 24 mm against* P. aeruginosa *bacterial strain. In this antibacterial assay, ciprofloxacin is taken as standard antibacterial agents. Antibacterial activity was also assessed in terms of MIC and MBC index of Hispidalin against selected bacterial strain and data are presented in Table 5. In the present study, Hispidalin peptide showed minimum MIC (80 *μ*g/mL) and MBC (100 *μ*g/mL) against* S. enterica* bacterial strain.

The inhibitory effect of Hispidalin against selected bacterial organisms is shown in Table 3. The Hispidalin exhibited significant antibacterial activity at 40 *μ*g/mL showing inhibition zone ranging from 18 to 29 mm in Figure 6. The tested Hispidalin peptide showed very convincing comparable antibacterial activity on most of selected gram-negative as well as gram-positive bacteria except* B. cereus* strain in comparison to standard antibacterial agent ciprofloxacin. This observation was defended based on earlier reports that* Bacillus *sp. strain has accumulated several plant origin peptides due to gene transfer between plant and bacteria. Due to this reason,* Bacillus *sp. has its endogenous peptide similar to plant peptide and adapted to the inhibitory actions of reported Hispidalin plant antimicrobial peptide [35]. Plant peptides carry out indispensable roles in plant survival, being directly involved in defense mechanisms against multiple pathogens. Amongst several classes of plant peptides like cyclotides, defensins, glycine-rich proteins, and other unusual classes, our identified Hispidalin falls under unknown unusual class of family, basic in nature with extended linear conformation without any disulfide linkage due to absence of any cysteine residue. In several reported antimicrobial peptides from seeds, where there are no similar structures, the main explanation of antimicrobial activity relies on the amphipathic surface of protein. Earlier studies have demonstrated that, although there are several antimicrobial peptides described, most of them inhibit only phytopathogens or only human pathogens [4, 8, 9]. Therefore, analyzing the antimicrobial peptides from plant sources described earlier in the literature [5, 12, 20, 31, 32, 36, 37], we can conclude that Hispidalin is the first peptide from* B. hispida* to present antibacterial activity against human pathogenic bacterium. The Hispidalin peptide sequence shows no homology to any previously described proteins and these findings are strengthened by the recent report from* Stellaria media* seeds showing antimicrobial activity without homology with previously known antimicrobial peptides from plant sources [5].

### 3.5. Antifungal Activity of Hispidalin

**Figure 7:**
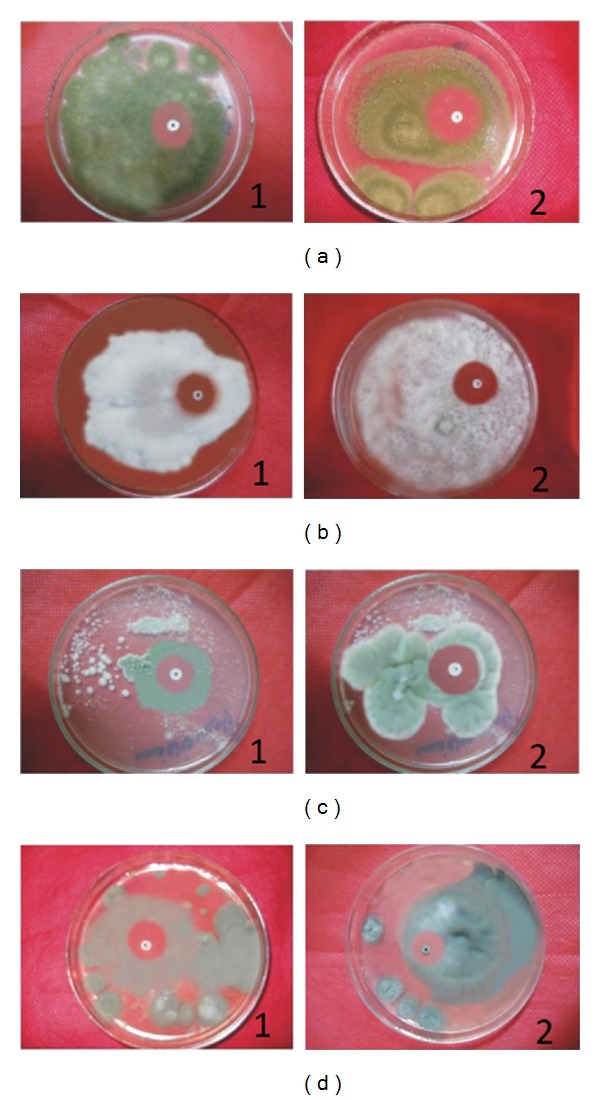
Inhibitory activity of Hispidalin (40 *μ*g/mL) on the growth of* A. flavus *(a),* F. solani *(b),* P. chrysogenum *(c),* C. geniculata* (d). by paper disk diffusion assay. Here, 1 is for peptide disc and 2 is for positive control griseofulvin antifungal agent.

The antifungal potency of the Hispidalin was studied on five different plant pathogenic fungi and compared to that of antifungal proteins griseofulvin in Table 4. The concentration of Hispidalin at 40 *μ*g/mL demonstrated significant zone of fungal hyphae growth inhibition ranging from minimum 18 mm (*C. geniculata*) to maximum 21 mm (*A. flavus*) (Figure 7 and Table 4). The comparative analysis of growth zone inhibition indicated that Hispidalin showed differential antifungal potential against different fungal strain; moreover, antifungal potency was highly comparable with well-known antifungal agent griseofulvin. Antifungal activity also assessed in terms of MIC and MFC index of Hispidalin tested against selected fungal strain is presented in Table 5. In terms of antifungal potential, Hispidalin demonstrated the least MIC (120 *μ*g/mL) and MFC (160 *μ*g/mL) against* A. flavus *fungal strain. The data clearly demonstrated significant antifungal potential of Hispidalin against most of selected plant and human pathogenic fungi except* C. gloeosporioides* fungal strain in comparison to standard antifungal agent griseofulvin. These fungal species have previously been shown to be responsive to a variety of antifungal proteins [11, 27, 29, 32, 35]. The inability of Hispidalin to act against* C. gloeosporioides* supports the reported claim that this fungal strain as an endophytic fungus has acquired parallel antifungal protein/peptide from the host plant [36]. The MICs value of Hispidalin against four fungal species ranged between 120 and 200 *μ*g/mL, which is highly comparable with those of other reported antifungal peptides such as* Udenya Africana* seeds peptides (MIC = 50–200 *μ*g/mL) [24], Lunatasin from* Phaseolus lunatus* (MIC and MFC = 50–200 *μ*g/mL), and castamollin from* Chinese chestnuts* (IC50 = 2.5 *μ*M) [23]. A further appraisal with reported data on the activity of other antifungal proteins/peptides reveals that Hispidalin is among the most potent antifungal proteins known previously.

### 3.6. Determination of Free Radical Scavenging Activity Using DPPH Assay

**Figure 8:**
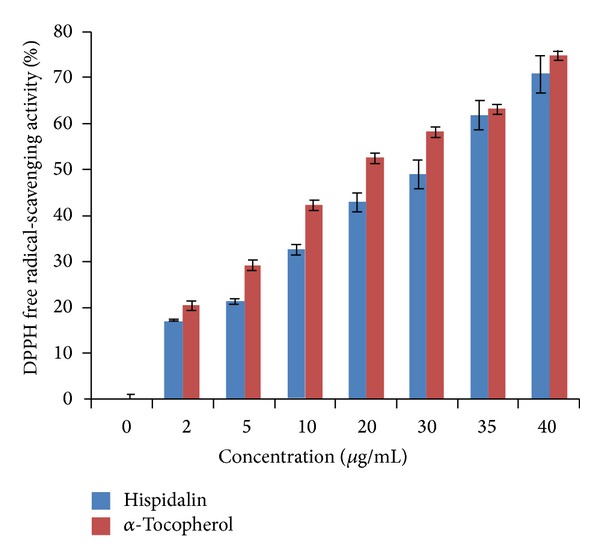
DPPH free radical scavenging activity of Hispidalin (0–40 *μ*g/mL). Data are the mean of triplicates ± SD.

As shown in Figure 8, the peptide showed a concentration dependent DPPH radical-scavenging activity and 40 *μ*g/mL of peptide showed maximum inhibition. DPPH radical-scavenging activity of the purified peptide was found to be 70.8% at 40 *μ*g/mL. The protein exhibited a statistically similar DPPH radical-scavenging activity when compared with standard antioxidant *α*-tocopherol which showed 74.9% scavenging of radicals at 40 *μ*g/mL concentration. The IC_50_ values of peptide and *α*-tocopherol are 14.84 *μ*g/mL and 14.15 *μ*g/mL, respectively. DPPH radicals, a stable radical used to evaluate the antioxidant activity of plant and microbial extracts [23, 24]. The obtained antioxidant activity presented in terms of free radical-scavenging activity is in agreement with previously reported protein/peptide from different plant sources [23–26]. The analysis of Hispidalin reveals the presence of three serine, three threonine, and five tyrosine amino acids containing hydroxyl group. Our findings support the previous claim that hydroxyl groups are responsible for free radical-scavenging activity of earlier reported peptides and flavonoids [24–26]. The amino acid analysis of Hispidalin also confirmed the hydrophilic nature and supports earlier suggestion that hydrophilic nature of peptide is contributing potent antioxidant activity against soluble free radicals like DPPH.

### 3.7. Lipid Peroxidation Inhibition Potential of Hispidalin Using Ferric Thiocyanate (FTC) TBA and TBA Method

**Figure 9:**
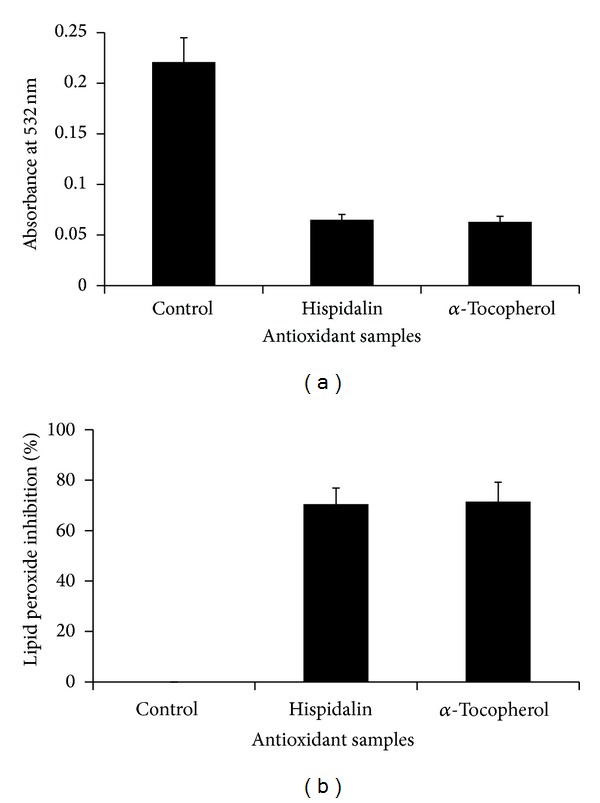
Antioxidant activity of Hispidalin (40 *μ*g/mL) in terms of lipid peroxidation. (a) Absorbance at 532 nm. (b) Percentage inhibition of lipid peroxide production. Data are the mean of triplicates ± SD.

FTC-TBA method measures the amount of peroxide value and products at the end of lipid peroxidation, where ferric ion was formed upon reaction of peroxide with ferrous chloride. The ferric ion will then unite with ammonium thiocyanate producing ferric thiocyanate, a red-colored substance. The TBA test combined with FTC is used to measure the secondary product of oxidation such as aldehyde and ketones [23, 24]. The data presented in Figures 9(a) and 9(b) shows the TBA mediated estimation of lipid peroxidation product MDA in the presence of Hispidalin and *α*-tocopherol at the seventh day of storage. The percentage of lipid peroxidation inhibition due to the peptide antioxidant activity was 69.5%, which is convincingly similar to *α*-tocopherol, where the percentage of inhibition was 71.5%. This result was in consonance with previous study [24–26] that among the different lipid peroxidation products used for the antioxidant assays, malonaldehyde MDA has been widely considered as a marker for the evaluation of antioxidant capacity of several chemicals and peptides. In several reports, lipid peroxidation inhibition due to antioxidant peptide is being measured by TBA-FTC method. Lipid is the principal component of cell membranes. A cell is seriously damaged by the lipid peroxidation. Hence, the inhibition of linoleic acid peroxidation is an important guide of antioxidant properties. The observed lipid peroxidation inhibition by Hispidalin may be attributed due to its metal ion chelating activity. The findings suggest that presence of hydroxyl group containing amino acids in Hispidalin may help in chelating ferrous ion leading to less lipid peroxidation products like aldehydes and ketones. In recent years, there are attention on the importance of bioactive peptide endowed with antioxidant activity and their significance in the pharmaceutical and therapeutic industry considerably promising substitute for the synthetic peptides. Based on the hydrophilic nature of Hispidalin and its ability to inhibit lipid peroxidation strengthen the earlier claim of lipid peroxidation ability of hydrophilic antioxidants.

## 4. Conclusion

In conclusion, we report purification of novel cationic antimicrobial and antioxidant Hispidalin peptide from* B. hispida* seeds. This peptide belongs to extended peptide conformation of plant AMP families. The peptides are active against human and plant pathogenic bacteria and fungi. The identification of this peptide holds good promise in the therapeutic and plant protection industry.

## Figures and Tables

**Table 1:** Antibacterial and antioxidant activity of FPLC and HPLC fractions.

Fractions	Zone diameter (mm) of fractions at 40 µg/mL concentration	% lipid peroxide inhibition by fractions at 40 µg/mL concentration	DPPH % inhibition at 40 µg/mL concentration
FPLC-P1	—	3.4 ± 0.3	17.8 ± 1.0
FPLC-P2	—	6.5 ± 0.6	19.6 ± 2.1
FPLC-P3	—	11.8 ± 1.0	12.1 ± 1.1
FPLC-P4	9	31.0 ± 2.7	18.9 ± 1.2
FPLC-P5	15	53.9 ± 6.0	64.1 ± 5.4
HPLC-P1	29	72.3 ± 6.82	76.83 ± 6.93
HPLC-P2	12	54.8 ± 7.83	48.93 ± 5.53
Standard	(Ciprofloxacin) 22	(*α*-Tocopherol) 64.76	(*α*-Tocopherol) 77.7

Data are represented as means ± SD.

**Table 2:** Purification table of Hispidalin showing percentage yield during FPLC and HPLC purification.

Name of fraction	Quantity of protein precipitate	Percentage yield
Ammonium sulphate precipitate	38 g out of 500 g dry seeds	7.6%
FPLC-P1	1.46	2.35%
FPLC-P2	1.94	9.7%
FPLC-P3	1.83	3.8%
FPLC-P4	2.23	11.75%
FPLC-P5	3.93	16.75%
HPLC-P1	0.56	56.73%
HPLC-P2	0.41	34.63%

**Table 3:** Antibacterial activity of Hispidalin (40 µg/mL) peptide using disc diffusion activity.

Bacterial strain	Zone diameter (mm) of peptide	Zone diameter (mm) of ciprofloxacin
*E. coli *	26 ± 2	29 ± 1.5
*P. aeruginosa *	24 ± 1.5	21 ± 2
*B. cereus *	—	—
*S. enterica *	29 ± 1	28 ± 1.5
*S. aureus *	25 ± 2	29 ± 2

Data are represented as means of triplicate ± SD.

**Table 4:** Antifungal activity of Hispidalin (40 µg/mL) peptide using disc diffusion method.

Fungal strain	Zone diameter (mm) of peptide	Zone diameter (mm) of griseofulvin
*A. flavus *	21 ± 1.5	30 ± 1
*F. solani *	21 ± 2	22 ± 1.5
*C. geniculata *	18 ± 2	22 ± 2
*C. gloeosporioides *	—	—
*P. chrysogenum *	20 ± 1.5	26 ± 1.5

Data are represented as means of triplicate ± SD.

**Table 5:** MIC and MBC/MFC of Hispidalin peptide.

Bacterial strain	MIC (*μ*g/mL)		MBC (*μ*g/mL)	
	Hispidalin	Ciprofloxacin	Hispidalin	Ciprofloxacin
*E. Coli *	100	30	120	40
*P. aeruginosa *	120	100	120	120
*B. cereus *	—	—	—	
*S. enterica *	80	40	100	60
*S. aureus *	100	50	120	60

Fungal strain	MIC (*μ*g/mL)		MFC (*μ*g/mL)	
	Hispidalin	Griseofulvin	Hispidalin	Griseofulvin

*A. flavus *	120	100	160	80
*F. solani *	130	120	180	60
*C. geniculata *	200	180	220	160
*C. gloeosporioides *	—	—	—	—
*P. chrysogenum *	180	100	160	80

*Mean of the MICs from three independent experiments. Hispidalin applied on the nutrient agar plates containing listed bacterial strains.
